# Deciphering the function of the fifth class of Gα proteins: regulation of ionic homeostasis as unifying hypothesis

**DOI:** 10.1007/s00018-024-05228-w

**Published:** 2024-05-10

**Authors:** Asmaa Abu Obaid, Ivan Ivandic, Sigrun I. Korsching

**Affiliations:** 1 Institute of Genetics, Faculty of Mathematics and Natural Sciences of the University at Cologne, Zülpicher Str. 47A, 50674 Cologne, Germany; 2 Present Address: Department of Optometry, Faculty of Modern Sciences, The Arab American University, Yousef Asfour Street, Ramallah, Palestine

**Keywords:** G protein-coupled receptor, Gnav, Osmoregulation, Signal transduction, Oogenesis

## Abstract

Trimeric G proteins transduce signals from a superfamily of receptors and each G protein controls a wide range of cellular and systemic functions. Their highly conserved alpha subunits fall in five classes, four of which have been well investigated (Gs, Gi, G12, Gq). In contrast, the function of the fifth class, Gv is completely unknown, despite its broad occurrence and evolutionary ancient origin (older than metazoans). Here we show a dynamic presence of Gv mRNA in several organs during early development of zebrafish, including the hatching gland, the pronephros and several cartilage anlagen, employing in situ hybridisation. Next, we generated a Gv frameshift mutation in zebrafish and observed distinct phenotypes such as reduced oviposition, premature hatching and craniofacial abnormalities in bone and cartilage of larval zebrafish. These phenotypes could suggest a disturbance in ionic homeostasis as a common denominator. Indeed, we find reduced levels of calcium, magnesium and potassium in the larvae and changes in expression levels of the sodium potassium pump *atp1a1a.5* and the sodium/calcium exchanger *ncx1b* in larvae and in the adult kidney, a major osmoregulatory organ. Additionally, expression of sodium chloride cotransporter *slc12a3* and the anion exchanger *slc26a4* is altered in complementary ways in adult kidney. It appears that Gv may modulate ionic homeostasis in zebrafish during development and in adults. Our results constitute the first insight into the function of the fifth class of G alpha proteins.

**Supplementary Information:**

The online version contains supplementary material available at 10.1007/s00018-024-05228-w.

## Introduction

G protein-coupled receptors (GPCRs) are among the largest gene families and a major pharmaceutical target for many human diseases and disorders. They are of central importance in cellular signalling, with trimeric G proteins transducing the signals from activated GPCRs into the cell interior. Gα subunits play a central role in these processes and are highly conserved. Throughout animal evolution the same five classes can be distinguished: Gs, Gi, Gq, G12 and Gv [1–3]. For all but one of these classes receptors, effectors and functions have been described [4], but the function of the most recently discovered fifth class, Gv, is unknown, even though it exists as a separate class already in the unicellular relatives of animals [1–3]. The phylogenetic relationship of Gv to the other 4 classes is not settled, but Gv may even be ancestral to all other G alpha classes [3]. Due to its presence in mostly aquatic species and its loss in tetrapods a function for aquatic organisms has been hypothesized [5]. In the vertebrate model organism zebrafish, the Gv class is represented by a single gene, *gnav1*, making zebrafish well-suited to a functional study of Gv.

The interaction of Gα proteins with receptors and effectors is mediated by class-specific conserved residues within the GTPase domain and the helical domain, but also within the C and N terminus [6, 7]. Those domains are differently conserved in Gv compared to the other four classes of Gα [5]. It is conceivable that Gv may have novel effectors beyond those described for the other four classes. Thus, it was impossible to derive clues from sequence analysis as to potential receptor and effector molecules of Gv.

Here we used a two-pronged approach to elucidate functions of Gv. Firstly, a thorough analysis of Gv expression in early embryos and larvae of zebrafish suggested several sites of action, among them osmoregulatory tissues. Secondly, we generated a zebrafish Gv mutant by CRISPR/Cas9-mediated frameshift and show several developmental defects in the mutant, which all appear to be related to a disturbance of ionic homeostasis. qPCR analysis identified significantly altered expression of several ion transporter genes thus allowing a first glimpse into the pathways and processes affected by Gv.

## Materials and methods

### Animal and tissue handling

Animal housing and maintenance was licensed by the office for environment and consumer protection of the city of Cologne, Germany. The experimental procedures were approved by the Federal ministry for nature, environment and consumer protection of Nordrhine-Westfalia, Germany, and were in accordance with the National Institutes of Health Guide for the Care and Use of Laboratory Animals (1996 revision). Zebrafish used in this study are of Ab/Tü genetic background and were raised in the local fish facility at 28 °C with a 14/10 photoperiod.

### Knockout of gnav1

To target the *gnav1* gene, 15 pg guide RNA was injected in one-cell stage zebrafish embryos together with 300 pg polyadenylated and capped Cas9 mRNA. Efficiency was evaluated by T7 endonuclease I assay at 1 dpf. Founders were raised, outcrossed, and germline transmission confirmed by T7 endonuclease I assay at 1 dpf. Littermates were raised to adulthood, and genotyped by sequencing. Of several frameshift mutations obtained, a 13b deletion event was selected to establish a line by repeated outcrossing followed by intercrossing to obtain homozygous knockout animals.

### In situ hybridisation

To obtain an informative view on Gv expression we selected six embryonic and larval stages, where a variety of morphogenetic changes occur: 6 h post fertilization (hpf), 12 hpf, 24 hpf, 2 days post fertilization (dpf), 3 dpf, and 5 dpf. Zebrafish were staged according to criteria outlined by [8]. Two different non-overlapping probes were used, with indistinguishable results. Sense probes were included for all stages and elicited no signal (Fig. S1). Whole mount in situ hybridisation was performed according to [9].

### Histological staining and morphological quantitation

The wildtype and mutant zebrafish larvae were raised in artificial freshwater, then sacrificed in ice water and fixed in 4% PFA in PBS overnight at room temperature. Acid-free double staining protocol (Alcian blue / ARS) was carried out as described [10] to stain cartilage and bone, respectively. The cartilage dimensions were measured as described by [11] using ImageJ [12].

### Quantification of ion content

Whole-body ion contents (calcium, magnesium, sodium, and potassium) were determined for pools of twenty-five wildtype and *gnav1*^*−/−*^ 5 dpf larvae raised in artificial freshwater using atomic absorption spectrometry as described by [13]. The ion content measurements were performed using inductively coupled plasma-optical emission spectroscopy (ICP-OES) spectrogreen (Spectro Ametek). To examine the influence of external calcium levels, pools of 15 embryos were raised in artificial fresh water (0.8 mM Na^+^, 0.16 mM Mg^2+^, and 0.3 mM K^+^) supplemented with 25 μM (low calcium), 0.25 mM (normal calcium) and 2.5 mM (high calcium).

### qPCR and endpoint RT-PCR

Freshly dissected adult kidneys and pools of 20 embryos/larvae (from 6 hpf to 5 dpf, raised in E3 medium) were sonicated in TRIzol for 10 s at 30 W (Bioruptor, Diagenode) for homogenisation. Isolation of total RNA was performed using TRIzol (Invitrogen) according to the manufacturer’s instructions. cDNA was synthesized using SuperScript IV First-Strand cDNA Synthesis System (ThermoFisher Scientific) according to manufacturer’s instructions. Endpoint RT-PCR was performed according to the manufacturer’s protocol using My-Budget PCR master mix (Bio-Budget), for primers used see Table S1.

RT-qPCR was performed according to the manufacturer’s protocol using primaQUANT qPCR-SYBR-Green master mix (Steinbrenner-Laborsysteme). No template control (NTC) and no reverse trancriptase (NRT) samples were included as negative controls. At least three biological replicates and two technical replicates of qPCR reactions were performed. qPCR data were obtained using the Bio-Rad CFX Manager software (Bio-Rad). For primers used see Table S1.

### Production of recombinant Gv protein for use in PRM (see below)

Gv protein was expressed from pOPIN-K with an N-terminal 6His-GST-tag using the primers listed in Table S1. *Escherichia coli* (Strain: Rosetta (DE3) pLysS) were transformed with the construct. Gv protein was expressed and purified as described by [14].

### Parallel reaction mass spectrometry (PRM)

Adult zebrafish were sacrificed in ice water; 3–4 adult kidneys were dissected, pooled and lysed in 30 μl RIPA buffer with 1x Protease inhibitor cocktail (Sigma Aldrich). Zebrafish embryos were raised in artificial fresh water (0.2 mM Ca^2+^, 0.8 mM Na^+^, 0.16 mM Mg^2+^, and 0.3 mM K^+^) until 48 hpf, sacrificed in ice water, and dechorionated by 1 mg/ml pronase (Sigma Aldrich) for 5 min, followed by a wash with 1x PBS. Dechorionated larvae were washed twice with Ringer solution, and deyolked by adding 10 mM EDTA/1x protease inhibitors/Ringer solution, followed by two washes with Ringer solution. Next, the embryos/larvae were lysed in 90 μl RIPA buffer mixed with 10 μl 10x protease inhibitor cocktail (Sigma Aldrich). Samples were homogenized with a probe sonicator for 10 s with 30 W on ice, incubated on ice for 20 min, and centrifuged for 30 min at 13,000 rpm at 4 °C. The supernatant was subjected to acetone precipitation. 50 μg precipitate or recombinant Gv protein were used for SDS-PAGE gel electrophoresis. A band of 40–50 kDa was cut and in-gel digestion with trypsin was performed (https://proteomics.cecad-labs.uni-koeln.de/protocols) as described, in parallel with Gv recombinant protein for calibration. The PRM assay itself was performed by the CECAD Proteomics Facility according to [15]. The samples were analyzed on a Orbitrap Exploris 480 (Thermo Scientific) mass spectrometer that is coupled to Ultimate 3000 HPLC System (Thermo Scientific). Raw data were processed by the CECAD Proteomics Facility using MaxQuant version 1.5.3.8 software.

### Statistical analysis

The significance was determined with two-tailed unpaired t-test or two-way ANOVA as indicated using GraphPad Prism version 9 for Windows, GraphPad Software, La Jolla California USA, www.graphpad.com. Error bars on the plots always indicate SEM. Significant *p* values are summarized with the following symbols: * 0.01 < *p* ≤ 0.05; ** *0.001 < p* ≤ 0.01; *** *p* ≤ 0.001.

## Results

### Gv pattern of expression suggests manifold functions in early development, but becomes more restricted during larval development, mostly to osmoregulatory organs

Zebrafish possess a single Gv gene, *gnav1*. Its expression was analysed during the first 5 days post fertilization using whole mount in situ hybridization. Embryonic stages examined were 6, 12, 24, and 48 h post fertilization (hpf), larvae were examined at 3 and 5 days post fertilization (dpf). Expression was examined with two different non-overlapping probes, which gave identical results. No signal was observed for sense probes (negative control) in any of the developmental stages analysed (Fig. S1).

**Fig. 1:**
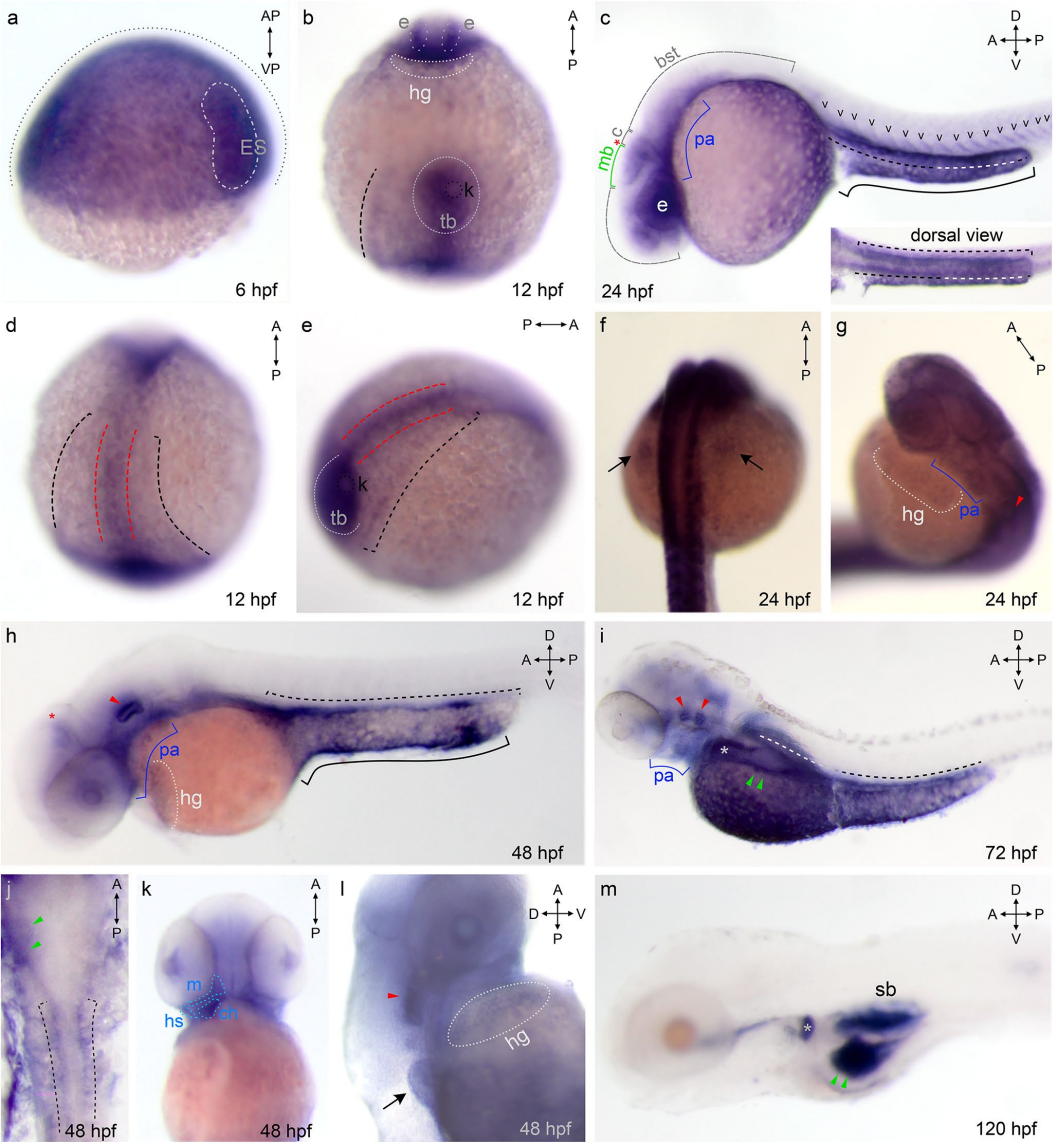
Increasingly more specific expression pattern of Gv suggests manifold roles during ontogenesis. Expression of Gv was examined by whole mount in situ hybridization in six developmental stages, 6, 12, 24, and 48 hpf (embryos), and 3 and 5 dpf (larvae). Organ abbreviations: bst, brain stem; c, cerebellum; ch, ceratohyal; e, eye; hg, hatching gland; ES, embryonic shield; hs, hyosymplectic; k, Kupffer’s vesicle; m, mandibular (Meckel’s + palatoquadrate); mb, midbrain; p.a., pharyngeal arches; sb, swim bladder; tb, tail bud. Symbols and line styles: white dotted line, hatching gland; white stippled/dotted line, hypoblast; red stippled line, notochord (axial mesoderm at 12 hpf); red asterisk, midbrain/hindbrain boundary; red arrow head, inner ear; green line, midbrain; green arrow head, gut; cyan stippled line, first and second pharyngeal arches (mandibular: Meckel’s cartilage and palatoquadrate and hyoid: ceratohyal and hyosymplectic); blue line, pharyngeal arches; gray dotted line, epiblast; gray asterisk, proximal convoluted tubule (PCT); black stippled line, pronephros (lateral plate mesoderm at 12 hpf); black line, yolk extension; black arrow, pectoral fin bud; black ‘v’, somites. For better visibility some lines are contrast adapted. **a**) 6 hpf; **b**), **d**), **e**) 12 hpf; **d**) dorsal view; **e**) dorsolateral view; **c**), **f**), **g**) 24 hpf; **c**) lateral view; **f**) dorsal view; **g**) dorsolateral view; **h**), **j**), **k**), **l**) 48 hpf; **h**) lateral view; **j**) dorsal view; **k**) ventral view; **l**) ventrolateral view; **i**) 72 hpf, lateral view; **m**) 120 hpf, lateral view

At the earliest stage examined, 6 hpf, gastrulation occurs, and the embryonic shield forms from blastoderm. Within the shield two layers can be distinguished, epiblast and hypoblast, giving rise to primitive ectoderm and mesoderm / endoderm, respectively. Gv is broadly expressed in both shield layers (Fig. 1a), suggesting involvement in these early differentiation processes. The Gv mRNA found in the gastrula might to some extent be of maternal origin, since some maternal transcripts are still present at 6 hpf in zebrafish [16].

In the early segmentation stage (12 hpf) Gv is found expressed in several organ primordia, including eye and brain primordia, lateral plate mesoderm (the pronephros progenitor cell field [17]), axial mesoderm (notochord rudiment), tail bud and in the forming hatching gland (Fig. 1b, d, e). In this stage Gv mRNA levels should reflect embryonic transcription, cf [18]. Within the tail bud Gv signal is present in tissues surrounding the Kupffer’s vesicle, which give rise to tail mesodermal derivatives, including notochord and muscle (Fig. 1b, e) [19]. Kupffer’s vesicle is a teleost-specific structure required for proper lateralization [20] and is not stained. In addition, broad staining along the anterior-posterior axis was observed (Fig. S1b).

At 24 hpf Gv is expressed in sensory and neuronal primordia (eye, ear, brain), in primordia of osmo- and iono-regulatory organs (pronephros and yolk syncytial layer, YSL), in primordia of cartilaginous structures (pharyngeal arches, pectoral fin bud), in somites and in the hatching gland (Fig. 1c, f, g). Expression in the brain is visible in forebrain, midbrain and hindbrain, in particular at borders such as the midbrain/hindbrain boundary, MHB (Fig. 1c). The pronephros develops from the Gv-positive lateral plate mesoderm, and shows strong Gv expression in pronephric tubule and pronephric duct (Fig. 1c). Distinct Gv expression is seen in the posterior YSL, in particular the YSL covering the yolk extension (Fig. 1c). The primordia of cartilaginous tissue including the pharyngeal arches region and pectoral fin bud are weakly stained (Fig. 1f-g). The expression in the somites shows the characteristic V-shaped myotome pattern and is more pronounced in the posterior (younger) somites. Together with the absence of somite staining in earlier (12 hpf) and later stages (48 hpf) this suggests a transient role for Gv in the maturation of myotomes. The hatching gland [8] is weakly stained (Fig. 1g).

At 48 hpf expression of Gv appears considerably more restricted, due to a pronounced decrease of Gv signal in brain, hatching gland, YSL of the yolk sac, and complete loss of detectable expression in eye and somites (Fig. 1h, l). The expression in pronephros and YSL covering the yolk extension remains pronounced (Fig. 1h, j). In otic vesicle, pectoral fin, and the pharyngeal arches region the expression of Gv intensifies (Fig. 1h, k, l). For the first time, bilateral symmetry is broken by a specific expression restricted to the left side of the embryo in the gut primordium region (Fig. 1j). The pharyngeal arches are much more well-defined and distinct than in the previous stage, both the more mature anterior and the less mature posterior arches express Gv similarly. Early precartilage condensations in the developing mandibular (Meckel’s cartilage and palatoquadrates) and hyoid arches (ceratohyal and hyosymplectic) express Gv (Fig. 1h, k). In the pectoral fin bud Gv expression is stronger in the center (Fig. 1l), i.e. the mesenchyme, the location of the first cartilage differentiating in the zebrafish embryo [8].

The next stage analyzed was 3 dpf. At this stage hatching is complete and organogenesis is nearly complete. Expression of Gv in the gut intensifies homogenously; expression in the YSL intensifies around the main yolk sac up to the level found in the yolk sac extension already at 2 dpf (Fig. 1i). Expression in the brain is further reduced. In several other organs, expression becomes restricted to subregions: in the otic vesicle to presumptive cristae and possibly anterior macula; in the pronephros mostly to the hook-shaped proximal convoluted tubule (PCT), in the pectoral fin to two parallel stripes at the border of the fin (Fig. S2a), most likely corresponding to actinotrichia forming cells. The collagenous actinotrichia fin rays are the first exoskeletal elements to form in the developing fin and express a fish-specific collagen, *col2a1b* [21]. Many other cartilaginous structures keep expressing Gv (branchial arches, in particular the ceratobranchial arches 1–3, Meckel’s cartilage, palatoquadrate, and ceratohyal cartilages, as well as the hyosymplectic, which is well defined at this stage) (Fig. 1i, Fig. S2a). Taken together, Gv expression is kept (in a more distinct pattern) in the otic vesicle and several osmoregulatory organs (pronephros, gut, and yolk). Gv expression is kept or extended in many cartilaginous tissues. Published expression patterns for 3 dpf [5] constitute a subset of the results reported here, presumably due to optimized sensitivity of detection in the current study.

At 5 dpf expression of *gnav1* is mostly confined to three organs: swim bladder, gut and the PCT of the kidney (Fig. 1m). The PCT harbors multiple loops and coils at 5 dpf, and the Gv expression faithfully mirrors these morphological specializations. The posterior segments of the pronephric tubule do not express *gnav1* at this stage (Fig. 1m, Fig. S1f).

In summary, *gnav1* expression begins broadly in early development (6–24 hpf) and becomes more and more tissue-specific in subsequent stages (2–5 dpf). *Gnav1* is transiently expressed *inter alia* in sensory tissues (12 hpf − 3 dpf), and cartilaginous tissues (24 hpf − 3 dpf), whereas it is expressed during the entire observation period in the kidney and the gut, both organs with a function in ion transport and thus osmoregulation. The hatching gland, a transient organ, expresses Gv mRNA during its entire existence. These patterns of Gv mRNA expression in the developing zebrafish implicate multiple roles for Gv in organogenesis. To identify such functions, we have generated a Gv knockout and analysed its embryonic and larval phenotype.

### Generation of a loss of function mutant for Gv

**Fig. 2:**
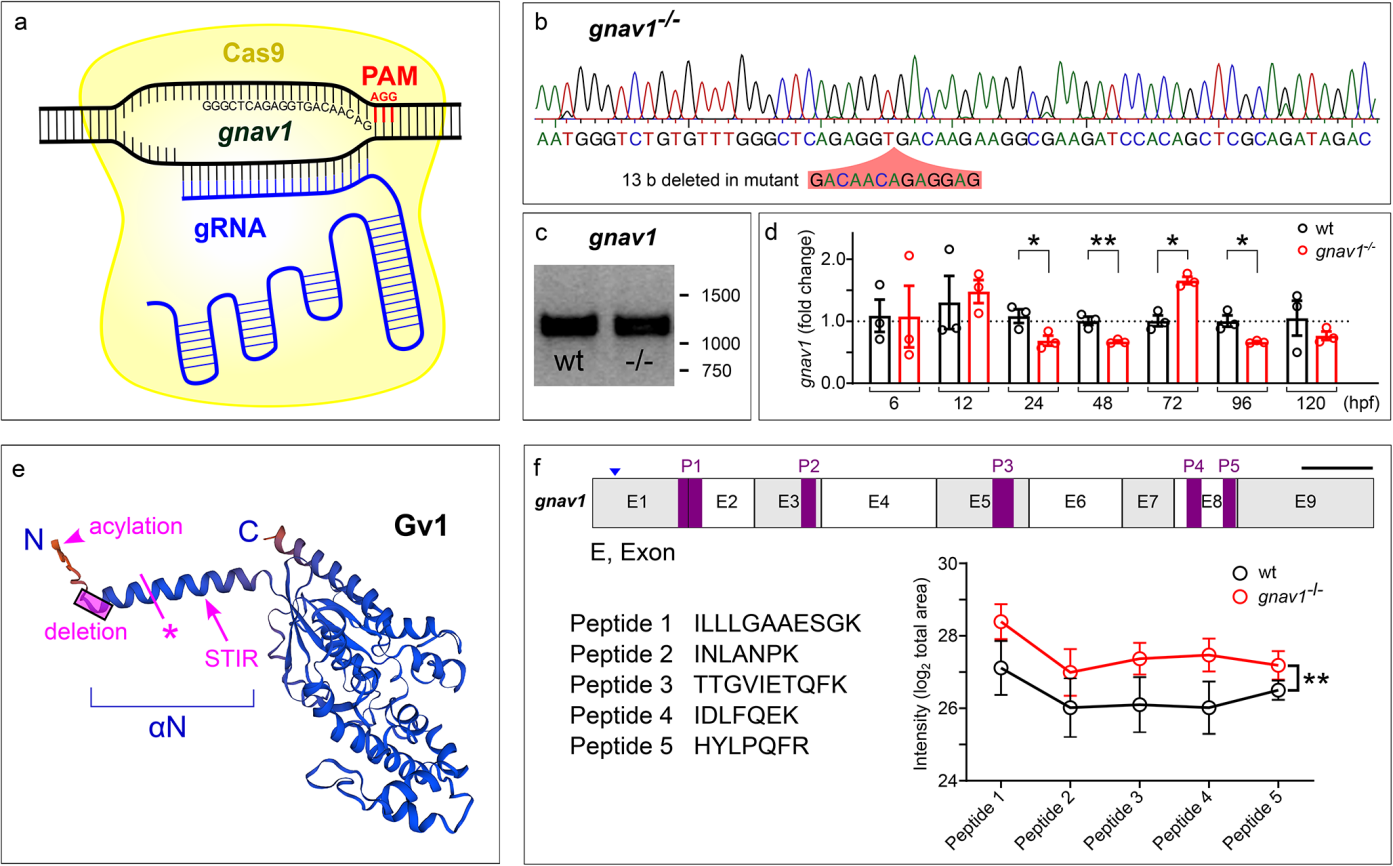
Knockout of zebrafish gnav1. (**a**) Schematic representation of knockout strategy using CRISPR/Cas9 method. The target sequence is shown. (**b**) Trace for homozygous knockout, with 13 b deletion indicated by red overlay in the nucleotide sequence. (**c**) RT-PCR for 5 dpf larvae shows presence of Gv RNA in the deletion mutant; 1% agarose TAE gel, stained with Midori green (NIPPON Genetics). (**d**) qPCR for Gv RNA in seven developmental stages. Wildtype (wt), black bars; deletion mutant, red bars. Significance estimated by two tailed unpaired t-test. *, *p* < 0.05; **, *p* < 0.01. y axis, fold change is normalized to reference genes rpl8 and rpl37. (**e**) Structure for Gv was taken from alphafold model AF-B0V3V7-F1-model_v4. The 13 **b** deletion is indicated by pink rectangle, it leads to a premature stop, pink asterisk. STIR, a potential secondary initiation site of translation. Acylation refers to two closely neighboring sites, for N-linked myristoylation and thio-palmitoylation (5). (**f**) Five peptides identified as suitable for parallel reaction mass spectrometry (PRM) are shown, together with their position in the exons of the gnav1 gene; the position of the deletion in exon 1 is indicated by arrowhead. Relative amounts present in 48 hpf larvae are shown for wildtype (black line) and deletion mutant (red line). Larvae are progeny of mutants and their wildtype siblings. The significance was determined with two-way ANOVA, **, *p* = 0.0055. Points represent mean+/-SEM of four independent determinations (each with a pool of 50 embryos)

A loss of function mutant for Gv was generated using CRISPR/Cas9 strategy [22]. A gRNA target close to the 5’ end of the coding sequence was chosen using an online algorithm http://crispr.mit.edu/ (Fig. 2a). The target sequence 5’-GGGCTCAGAGGTGACAACAG-3’ (sense) begins 11b after the start codon and lies in the first exon. gRNA and Cas9 mRNA were co-injected in one cell stage zebrafish embryos, which were raised to adulthood, genotyped and examined for germline transmission by outcrossing to wildtype. Several deletions (-2, -3, -4, -7, -12, -13, -18) and one insertion (+ 7) were observed, five of which resulted in frame shifts. The 13b deletion was selected to establish a transgenic line by repeated outcrossing and intercrossing (Fig. 2b). This deletion results in a premature stop codon in the N-terminal helix of Gv, i.e. in a short fragment missing all known domains of Gv (*cf* [5]). The mutation was validated by sequencing of genomic DNA in each generation (Fig. 2b).

To predict potential off-target sites in the mutant, we employed an algorithm [23] as implemented on http://crispr.mit.edu/. The selected gRNA possessed only low quality potential off-target sites with 3 or more mismatches. None of these off-target sites was present on the same chromosome as *gnav1* (chromosome 22). The top scoring three potential off-target sites were not positioned in coding regions. Nevertheless, we analysed all three sites in the heterozygous F1 generation. After amplification of genomic DNA surrounding the sites T7 endonuclease 1 digest was performed. No evidence for any mutation in these sites was found (Fig. S3). Furthermore, phenotypic analysis only was begun after repeated outcrossing of the mutants. Phenotypes were consistent over several generations. As indicated in the individual experiments, phenotypes were compared between homozygous *gnav1* mutants and their wildtype siblings to ensure highest possible similarity of genetic background for mutants and wildtype.

We first examined whether Gv RNA would be subject to nonsense-mediated decay (NMD) in the deletion mutant. RT-PCR showed Gv RNA levels in the mutant similar to wildtype levels (Fig. 2c). A closer inspection by qPCR showed some quantitative changes in the mutant Gv RNA levels during development: significant reduction at three developmental stages (1, 2, and 4 dpf) and a significant increase at 3 dpf (Fig. 2d). While the decreases conceivably could be caused by inefficient NMD, the increase at 3 dpf might point to compensatory Gv expression possibly due to a feedback loop.

Next, we analysed Gv protein levels in the mutant. However, mass-spectrometric analysis of pools of larvae was insufficiently sensitive to detect Gv in the wildtype (several Gα proteins from the other four classes were detected). Therefore, we turned to parallel reaction mass spectrometry (PRM), a more sensitive method. Recombinant Gv was subjected to PRM to identify suitable peptides. Eleven peptides were obtained, of which five were unique to Gv and not shared with other Gα proteins. All five peptides were not only found in pools of wildtype larvae, but also in the mutant (Fig. 2f). This unexpected result suggests the use of a secondary translation initiation site (STIR) in the deletion mutant. The first peptide, ILLLGAAESGK, spans the exon1/exon2 border and is present at similar levels compared to the other peptides (Fig. 2f). Thus, secondary initiation should occur somewhere between the premature stop codon and peptide 1, with L28 being a possible candidate (Fig. 2e).

The resulting mutant protein is missing most or all of the N-terminal αN helix, in particular the acylation sites, but may be C-terminally complete (Fig. 2e). The αN helix binds to the GPCR and transmits the conformational changes of the activated receptor to the nucleotide-binding pocket of the Gα subunit [6, 7]. Deletion or truncation of this vital motif likely disables the activation of Gv, thereby interrupting Gv-mediated signal transduction. Moreover, the effective concentration of the truncated Gv at the plasma membrane is expected to be much smaller due to the absence of acylation sites. Acylation is conserved in all G alpha proteins [6] and mutation of the acylation site has been shown to destroy their membrane association [24]. Interestingly, the mutant protein is present at higher levels compared to wildtype (Fig. 2f), possibly due to a feedback loop. All in all, it appears highly likely that the truncated Gv is not functional. Next, we proceeded with functional analysis of the Gv mutant.

### Reduced oviposition in the Gv mutant

**Fig. 3:**
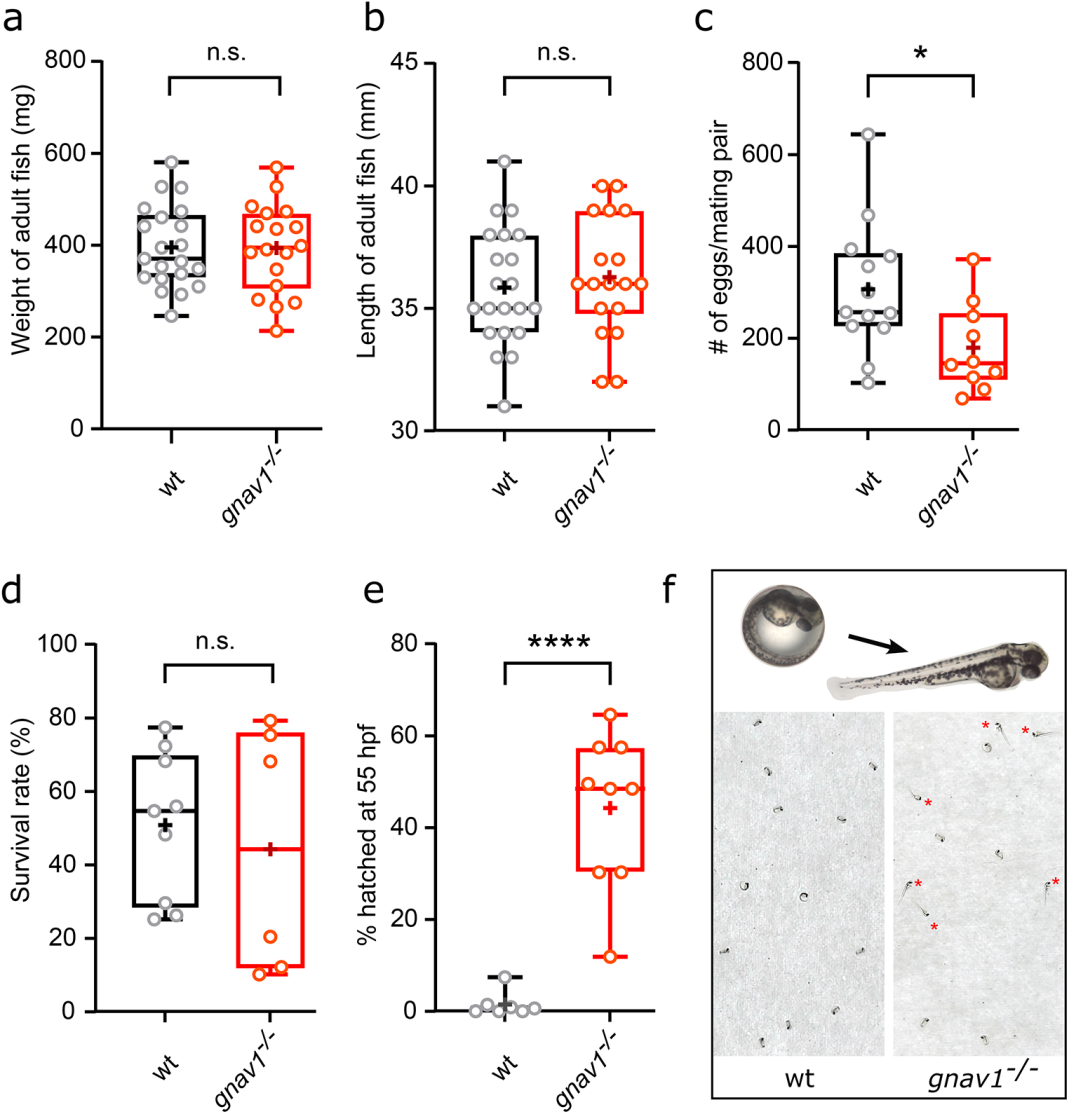
Reduced oviposition and premature hatching in Gv knockout. Wildtype, wt; gnav1 deletion mutant, gnav1^−/−^. Data for panels a) to e) are shown as whisker plots with quartile segments, mean values are indicated by crosses, individual measurements are shown as circles. (**a**) No difference in weight between 15 months adult wildtype and their homozygous gnav1 deletion mutant siblings. (**b**) No difference in body length between 15 months adult wildtype and their homozygous gnav1 deletion mutant siblings. (**c**) Clutch size (number of fertilised oocytes) is reduced in the homozygous gnav1 deletion mutant compared to their wildtype siblings exposed to the same mating schedule (9-month-old experienced breeding pairs, *n* = 10–13, *p* < 0.05). Unfertilized oocytes were rare. (**d**) No difference in survival rate at 24 hpf between wildtype and the homozygous gnav1 deletion mutant (sibling parents). After 1 dpf nearly no further death was observed for wildtype and mutant. (**e**) Hatching was evaluated early in the third day (54–56 hpf) and is faster in the homozygous gnav1 deletion mutant compared to wildtype (sibling parents) (*p* < 0.0001). Each circle represents a separate clutch. (**f**) Representative photographs of wildtype and gnav1 deletion mutant at 55 hpf, top pictures show a magnification for an unhatched embryo inside the chorion and a hatched fish. Red asterisks point out hatched larvae. Significance was estimated by two tailed unpaired t-test

Clutch size of adult homozygous knockouts was strongly reduced compared to progeny from wildtype siblings (Fig. 3c). However, no embryonic lethality above control values was observed, thus the viability of the embryos was not affected (Fig. 3d). Furthermore, general behavior and gross morphology of adult homozygous knockouts appeared unchanged compared to wildtype. Also, length and weight of adult homozygous knockouts were not significantly different from wildtype controls (Fig. 3a, b). Thus, the effect on oviposition might result from a specific defect in oogenesis. Consistent with this hypothesis, transcriptome studies show expression of Gv in the ovary and, particularly high, in unfertilized oocytes (Fig. S4).

### Premature hatching in the Gv mutant

Wildtype zebrafish hatch throughout the third (and fourth) day post fertilization [8]. We observe consistently that large numbers of Gv mutant fish already hatch early in the third day, when wildtype fish are still overwhelmingly inside the chorion (Fig. 3e, f). This effect is highly significant (*p* < 0.0001). In principle, premature hatching could be caused by three different processes or a combination thereof: (a) lesser mechanical stability of the chorion synthesized in mutant larvae, (b) increased motility of the larvae inside the chorion, and (c) premature or excessive production of hatching enzyme, which softens the chorion. We do not see any difference in motility between mutant and wildtype larvae, excluding the second possibility. However, when 48 hpf larvae were agitated by pipet transfer (during collection), we noticed frequent acute rupture of the chorion of mutant larvae. This never happened with wildtype larvae, suggesting reduced mechanical stability of the chorion in mutant larvae. The specific expression of Gv in the hatching gland suggests an involvement of this gland in this phenotype supporting (c), possibly together with (a). Since the absence of functional Gv in the hatching gland results in premature hatching, one may expect Gv to have a negative regulatory influence on hatching enzyme synthesis, activation or release (the enzyme is stored in granules in the hatching gland cells).

### Craniofacial abnormalities in the Gv mutant

Juveniles with the mutation appeared outwardly very similar to wildtype, but seemed to be more fragile. To investigate this, we employed Alcian Blue to label acidic glycoproteins, which are a major component of cartilage, and Alizarin Red S (ARS) to assess bone mineralization. Progeny from homozygously mutant Gv and sibling wildtype fish was stained and photographed in parallel. Staining was analysed for 3, 5 and 11 dpf larvae (brightfield for 3 and 5 dpf, fluorescence for 5 and 11 dpf).

**Fig. 4:**
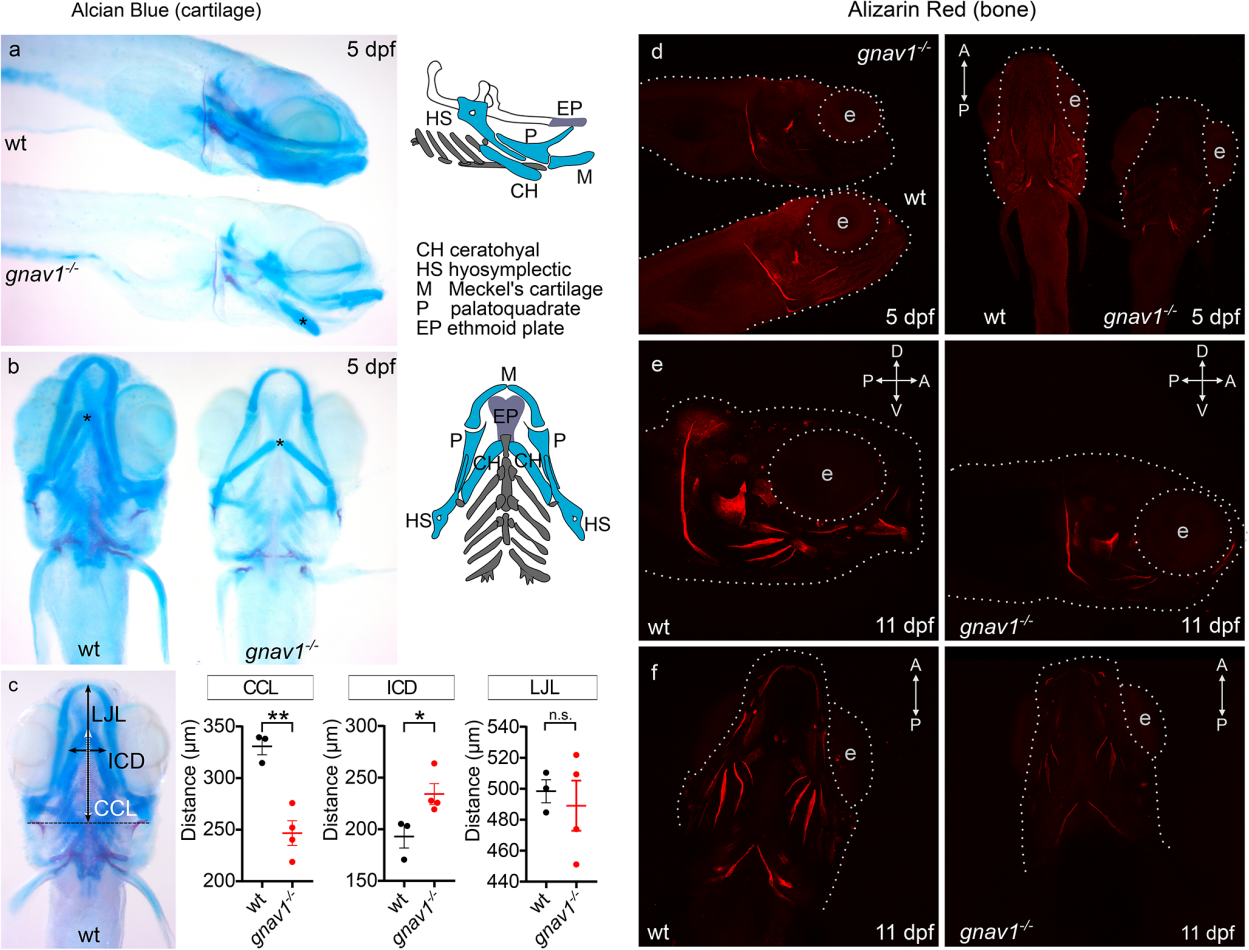
Craniofacial abnormalities in the *gnav1* deletion mutant. Cartilage of wildtype (wt) and *gnav1* deletion mutant (*gnav1*^−/−^) 5 dpf larvae (from sibling parents) was stained with Alcian Blue (panels **a**, **b**, **c**). Mineralization of bone structures was visualized with ARS fluorescence at 5 dpf (panel **d**) and 11 dpf (panels **e**, **f**). Note the clear reduction in staining intensity for the mutant for both dyes. (**a**) Lateral view, anterior is to the right, the scheme color-codes the cartilage visible in this orientation (scheme was modified from [25]. Asterisk, ceratohyal cartilages. Wildtype (wt) and mutant (*gnav1*^−/−^) were stained and photographed side by side. (**b**) Ventral view, anterior is up, the scheme color-codes the cartilage visible in this orientation (scheme was modified from [25]. Asterisk, ceratohyal cartilages. **c**) Left micrograph, definition of three axial distances, ceratohyal cartilage length, CCL; intercranial distance, ICD; lower jaw length, LJL. The quantitation for CCL, ICD and LJL shows mean, 1st and 3rd quartile as well as the individual data points for wildtype (black) and *gnav1* deletion mutant (red). Significance estimated by two tailed unpaired t-test, *p < 0.05, **p < 0.01, and ns = not significant. Error bars denote SEM. **d**) 5 dpf larvae were stained and photographed side by side. Left micrograph, lateral view, anterior to the right; right micrograph, ventral view. Gray dotted lines outline head region and the eye (**e**). **e**), **f**), 11 dpf larvae needed to be photographed singly, due to size; exposure time identical for wildtype and mutant. **e**) 11 dpf larvae, lateral view, genotype as indicated. f) 11 dpf larvae, ventral view, genotype as indicated

Alcian Blue staining was decreased in the mutants compared to wildtype in both stages analysed, 3 and 5 dpf (Fig. 4a, b, Fig. S2b). Identifiable cartilage structures such as ceratohyal and ethmoid plate showed clearly reduced staining intensity. Furthermore, staining was reduced homogenously throughout the body. This suggests that extracellular matrix deposition is generally impaired in the Gv mutants, which would negatively impact mechanical stability.

Moreover, we noticed different angles and distances of some craniofacial cartilage anlagen in mutant fish, among them a shorter, broader and ventrally displaced ceratohyal (a component of the lower jaw) (Fig. 4a, b). We therefore proceeded to quantify three accurately measurable distances, CCL (ceratohyal cartilage length), ICD (intercranial distance) and LJL (lower jaw length). We find that the lower jaw is deformed in mutant zebrafish larvae, as seen by a significant increase in ICD and a significant decrease in CCL (Fig. 4c).

Next, we examined bone mineralization using ARS. This dye stains cations (mainly calcium). We observe severe reduction of mineralization at 5 dpf (Fig. 4d) and 11 dpf (Fig. 4e, f) in the developing bone structures including vertebra (Fig. S2c). At 3 dpf bones are not formed yet [26] but the wildtype shows diffuse mineralization in head, yolk sac, pronephros, and gut, whereas these regions show extremely reduced staining intensity in the mutant (Fig. S2b).

### Both ion levels and ion transporter expression are altered in the Gv mutant

**Fig. 5:**
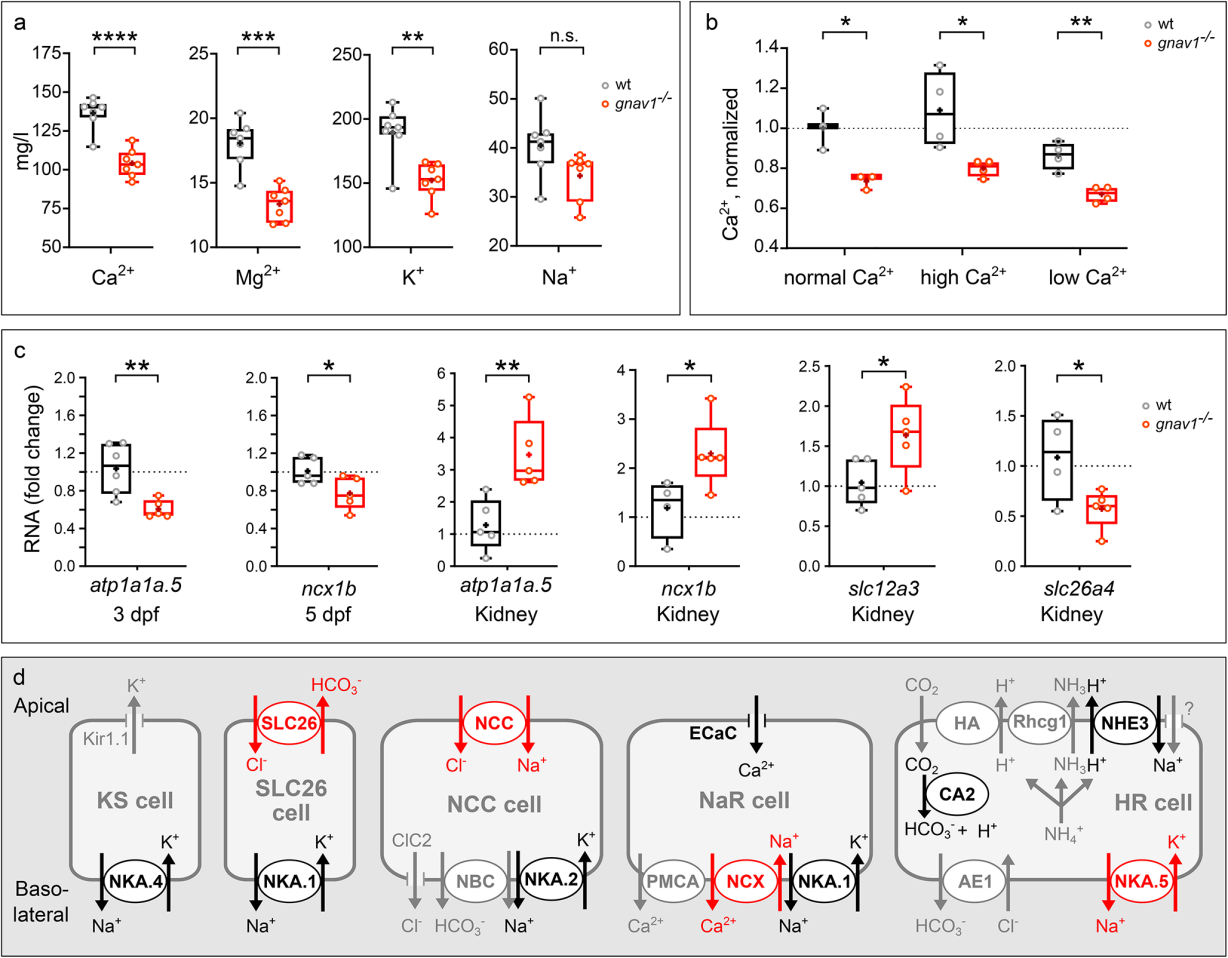
Decreased cation levels and altered ion transporter gene expression in Gv mutant zebrafish. Data for panels **a**) to **c**) are shown as whisker plots with quartile segments, mean values are indicated by crosses, individual measurements are shown as circles. (**a**) Cation levels were analysed by atomic absorption spectroscopy in pools of 25 larvae from sibling parents (5 dpf), *n* = 7 biological replicates. Note that all levels decrease in the mutant compared to wildtype (wt), three of them significantly. (**b**) A decrease in calcium levels in the mutant persists both in high and in low calcium rearing. Calcium levels are normalized to wildtype in normal calcium, *n* = 3–4 biological replicates. (**c**) qPCR of 3 dpf and 5 dpf larval pools (20 larvae, Gv mutant and wildtype from sibling parents) and adult kidneys of Gv mutants and their wildtype siblings showed altered gene expression as indicated, 4–5 biological replicates. Note that mutant adult kidney appeared phenotypically normal and showed no signs of edema. **a**, **b**, **c**) Significance was estimated by two tailed unpaired t-test: *, *p* < 0.05, **, *p* < 0.01, ***, *p* < 0.001; ****, *p* < 0.0001. (**d**) Current understanding of ion transporters, channels and exchangers in zebrafish ionocytes is depicted schematically (scheme modified from [27]. A largely overlapping set of genes is present in kidney [28]. Red ovals and arrows, significant alteration of expression was seen in the Gv mutant. Black ovals and arrows, no significant changes; gray ovals and arrows, not tested. Protein names are given, NKA.5 refers to gene *atp1a1a.5*; SLC26 refers to gene *slc26a4*; NCC refers to gene *slc12a3*, NCX refers to gene *ncx1b*

All larval phenotypes of the Gv mutant fish described above exhibit potential links to altered ionic homeostasis. If indeed Gv is involved in regulating ionic homeostasis we would as a consequence expect an altered ion composition of mutant fish. We used atomic absorption spectroscopy to measure the total amounts of the main monovalent and divalent cations, Na^+^, K^+^, Ca^2+^, Mg^2+^, in 5 dpf larvae of wildtype and mutants. 25 larvae were pooled for each determination and seven measurements were made for each genotype and ion. Dry weight and wet weight were nearly identical between wildtype and mutant larvae (Fig. S5). In mutant larvae a significant decrease was observed for K^+^, Ca^2+^, and Mg^2+^ (Fig. 5a). The calcium decreases persisted independent of external calcium levels (Fig. 5b). These data suggest that Gv is involved in signalling pathways regulating ionic homeostasis.

Next, we examined expression levels of ionoregulatory genes such as ion transporters and ion channels (Fig. 5c). Ionic homeostasis in larval zebrafish is regulated by kidney and five different types of ionocytes in skin [27], which differ by their set of ion transporters and channels (Fig. 5d). In the adult fish, gills and kidney are the main osmoregulatory organs, which express a largely overlapping set of ion transporters and channels [28]. We used qPCR to analyse expression levels of nine such transporters and the *ecac* ion channel in adult kidney, 3 and 5 dpf larvae. For larvae we normalized to two reference genes, *rpl8* and *rpl37*, for adult kidney four reference genes were used (*elf1*, *b2m*, *bactin*, and *rpl8*). Of all genes analysed (see Table S2 for the complete list) we observe significant differences in expression between wildtype and Gv mutant for four ion transporter genes, *atp1a1a.5, ncx1b, slc12a3*, and *slc26a4* (Fig. 5c). Increases up to threefold and decreases down to one half were observed (Table S2). No significant changes were seen for three other Na^+^/K^+^ ATPases (NKA.1 and NKA.2, and NKA.4), a calcium channel (ECaC) and a sodium/proton antiporter (NHE3) (Table S2).

Taken together we found both altered ion levels in the Gv mutant and altered expression of some ion-regulating genes. This suggests that Gv is indeed involved in regulating ionic homeostasis.

## Discussion

Biological processes influenced by Gv include oviposition, hatching, and craniofacial development. Pleiotropic effects such as these are characteristic for knockouts of other G alpha proteins [29, 30]. The current data do not allow to distinguish unambiguously, to what extent these phenotypes are caused by the absence of active Gv in the affected organs, by remote action of Gv in other organs, or by disturbance of developmentally earlier processes. It also can’t be excluded that the truncated Gv might act as scavenger for beta/gamma subunits, cf [31], although the missing alphaN helix would remove part of the binding interface to beta/gamma subunits [6]. However, the specificity of the observed phenotypes together with co-localisation of Gv expression in the affected structures suggests that oviposition, hatching, and cartilage phenotypes may be caused by a local action of Gv in these tissues. All these processes can be disturbed by abnormal ionic homeostasis, and indeed Ca^2+^, Mg^2+^, Na^+^ and K^+^ levels are decreased in mutant larvae, all but Na^+^ significantly so. Moreover, four ion transporter genes show significantly altered levels of expression in the Gv mutant.

*The oviposition phenotype, a strongly reduced clutch size of Gv mutants*, was not accompanied by a decrease in the viability of the progeny, thus this effect might result from a defect in oogenesis, either in maturation or ovulation. Indeed, transcriptome studies have shown the presence of Gv in the ovary and in particular in the unfertilized oocytes (Fig. S4 [32]). Ovulation appears to be regulated by nuclear receptors [33], but maturation requires a GPCR, mPRα, an oocyte progestin receptor [34, 35]. Thus, mPRα seems to be a good candidate for a Gv activator molecule and could be examined in this respect in follow-up studies.

**Fig. 6:**
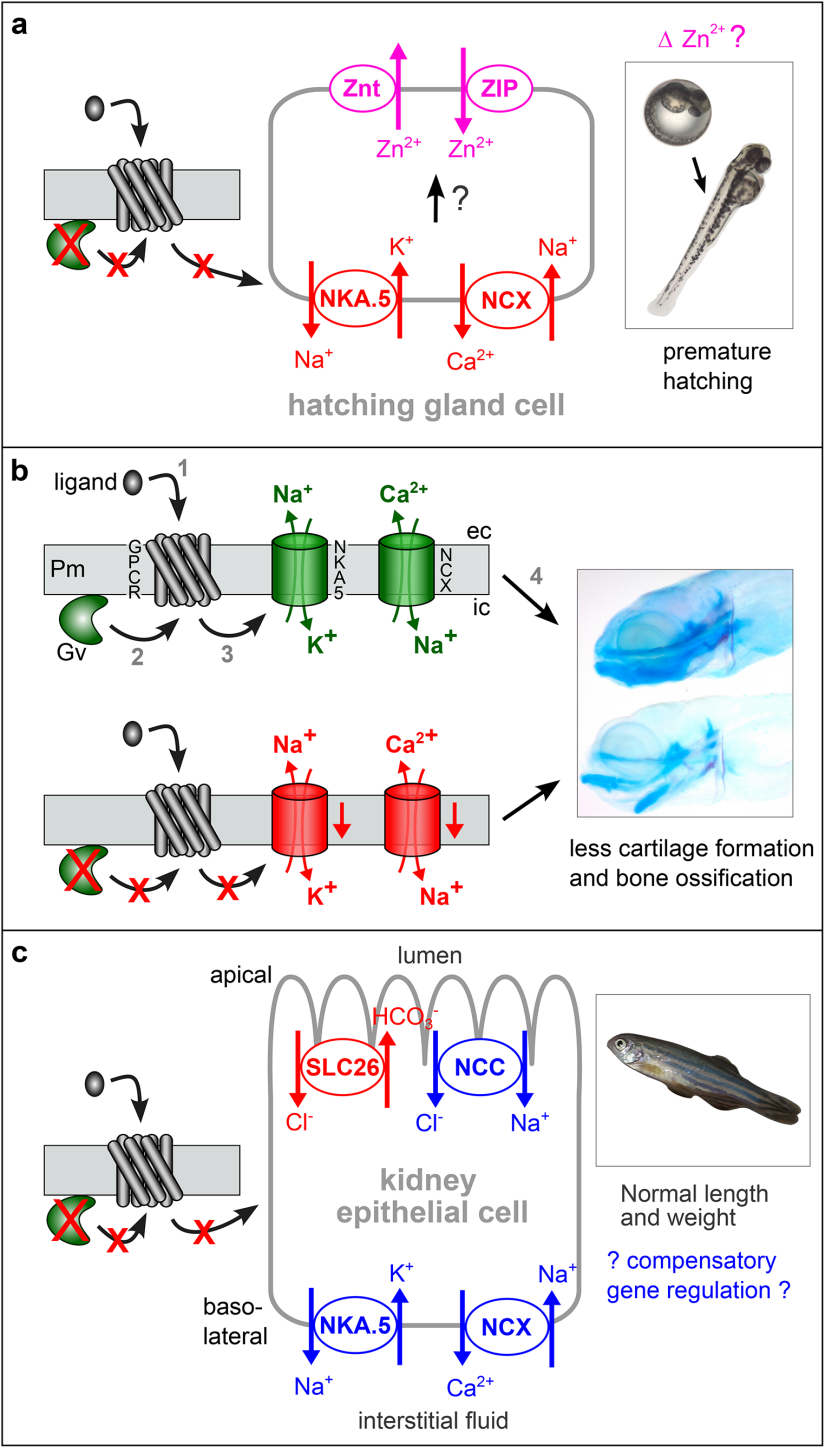
Hypotheses for Gv signalling pathways in three developmental stages. Arrows may represent several molecular steps. Red color indicates block (red crosses) or decrease in the mutant, blue depicts an increase, green represents wildtype levels, magenta shows hypothetical effect. (**a**) In the mutant embryo decreased levels of NKA.5 (Na^+^/K^+^ ATPase) and NCX (NCX1b) might secondarily influence Zn^2+^ levels in the hatching gland, possibly via two zinc transporters present in hatching gland cells [39], which would influence hatching enzyme activity, leading to premature hatching. (**b**) Top row shows wildtype situation: Gv is activated by so far unknown ligand/receptor pairs and eventually regulates NKA.5 and NCX expression, which is required for proper cartilage formation (upper zebrafish larva). Pm, plasma membrane; ec, extracellular; ic, intracellular; numbers refer to temporal order. Bottom row shows mutant situation, red crosses depict losses of the respective elements; red arrow, downregulation of expression of Na^+^/K^+^ ATPase and NCX may lead to reduced and distorted cartilage formation (lower zebrafish larva). Note that reduced calcium extrusion means less calcium getting inside the animal via the basal surface of ionocytes. For details of larval micrographs see Fig. 4b legend. (**c**) In the mutant adult kidney four ion transporters act to some extent compensatory: A decrease in SLC26 may be balanced by an increase in NCC for the chloride level, and sodium levels are reduced by increased NKA.5, but augmented by increased NCC and NCX. Such compensatory effects may explain the absence of outwardly visible phenotype in adult mutant zebrafish (framed inset)

*The earliest embryonic phenotype is drastically accelerated hatching in Gv mutants*. Several environmental stressors influence the hatching time in wildtype zebrafish, e.g. high salt accelerates hatching about a day [36], similar to the amount of acceleration we observe. Thus, it is conceivable that the altered ion levels observed in the Gv mutant might play a role in the accelerated hatching phenotype. The hatching process involves production, activation and release of zinc-dependent metalloproteases by cells of the hatching gland [37], which soften the chorion by cleaving its main components, zona pellucida proteins [38]. Zinc inside the hatching gland cells is essential for hatching and its levels are regulated by specific transporters [39]. The altered ionic homeostasis in the Gv mutant might encompass altered Zn^2+^ levels. Since Gv is expressed in the hatching gland throughout its existence (12 hpf to 48 hpf [8]), Gv could exert an inhibitory effect either on expression or release of these metalloproteases or on the expression of the transporters required for their activation, possibly *via* Gv’s effects on ionic homeostasis (Fig. 6).

*The craniofacial phenotype of the Gv mutant consists of strong decreases in ECM deposition and bone mineralization as well as altered cartilage morphology*. The cartilage marker we used, Alcian Blue, stains proteoglycans which are an essential component of cartilage and more generally ECM. Defects in proteoglycan synthesis lead to pleiotropic cartilage malformation and fragility in zebrafish [40] and humans [41], very similar to the phenotypes we observe here, down to an increased ceratohyal angle in a proteoglycan synthesis mutant [40]. The reduced proteoglycan levels of the Gv mutant may be caused by an altered balance between synthesis and degradation. It is well known that this balance is regulated by ion levels surrounding the chondrocytes which build (and degrade) the cartilage (for a review see [42]). Ion levels also seem to influence the differentiation of chondrocytes [43], which is another pathway whose disregulation results in cartilage (and bone) dysplasia. We hypothesize that the dysplasia, tissue fragility, and overall reduced Alcian Blue staining of the mutant larvae result from ionic homeostasis defects that impact the ECM protein metabolism (Fig. 6).

The massive decrease in bone mineralization seen in the Gv mutant is most likely a direct result of the decreased ion levels in the mutant larvae, in particular calcium and magnesium. These ions stimulate bone formation [44], their decrease is associated with skeletal defects, and they are the main cations stored in zebrafish bone [45]. Thus, the impaired bone ossification in the Gv mutants is expected to occur as a result of deficiency in these minerals due to impaired function of the osmoregulatory organs. Consistent with such a remote effect we do not detect Gv expression in bone by in situ hybridisation.

A somewhat similar craniofacial phenotype is observed for *gnai3* mutations in humans [46]. We note that bone defects (and reduced fertility) have been observed for a loss-of-function mutation of the Wnt-interacting GPCR *lgr4* [47, 48], which exhibits a strikingly similar expression pattern during development compared to Gv (this manuscript and [49]). Further studies will show, whether Lgr4 might signal through Gv.

*The major osmoregulatory organ kidney shows significant changes in gene expression for the Na*^*+*^*/K*^*+*^*ATPase NKA.5, Na*^*+*^*/Ca*^*2+*^*exchanger 1b (ncx1b), the cotransporter NCC (slc12a3) and the anion exchanger pendrin (slc26a4)*. As a fresh water fish, zebrafish need to actively absorb ions to maintain ionic homeostasis, and the electrochemical gradient for ionocytes and kidney cells powering such ion uptake is generated by Na^+^/K^+^ ATPases situated in the basal membrane of these cells. Thus, the Na^+^/K^+^ ATPases occupy a central position in influencing directly or indirectly all ionic gradients across the plasma membrane. We observe a clear increase of NKA.5 in the Gv mutant in the adult kidney, but an opposing trend in mutant larvae. Likewise, the sodium/calcium exchanger ncx1b shows an increase in the adult kidney of the mutant, but a decrease in mutant larvae. *ncx1b* is responsible for transepithelial Ca^2+^ uptake [50], the decreased expression of *ncx1b* in larvae could therefore contribute to the decreased calcium levels observed by direct ion measurement and by ARS staining for bone calcification. However, it is not expected to be the sole contributor, since decreased calcium levels in mutant larvae are observed also in high external calcium conditions and are not more severe in low external calcium.

Why does adult kidney show opposing trends to those observed in larvae? An increase in larval kidney could be dominated by a larger decrease in another organ, since in larvae *atp1a1a.5* and *ncx1b are* expressed in several organs, not only in kidney [51, 52]. See [13] for opposing effects on NKA.5 expression in kidney vs. gills in zebrafish larvae. It is also conceivable that the increased expression in adult kidney might represent a compensatory mechanism (Fig. 6).

NKA pump subunits are highly expressed in human chondrocytes, and regulate chondrocyte differentiation and bone ossification in mice [53, 54]. NKA may regulate ECM homeostasis by modulating the expression of collagens and proteins associated with ECM degradation [54]. Furthermore, NKA.5 and NCX1 do play a role in calcium homeostasis [54, 55]. Taken together, this suggests that the NKA.5 and NCX1 deficiency in *gnav1*^*−/−*^ mutant larvae could be the reason for the craniofacial defects that we observed (Fig. 6).

The cotransporter NCC (*slc12a3)* and the anion exchanger pendrin (*slc26a4*) both serve to (re)absorb chloride ions into ionocytes and renal tubular cells [27, 56, 57]. Beyond these direct effects, many other ion levels are influenced by mutations of these genes. Hypokalemia, hypomagnesemia, and hypocalciuria are observed for human NCC loss-of-function mutations [58]; pendrin deletion in mice leads to loss of calcium re-absorption [59]. NCC and pendrin show opposing changes in roughly similar magnitude in adult kidney of mutant fish, thus it is conceivable that these changes are compensatory for each other, as has been shown for mouse kidney [60]. Interestingly, NCC and NKA.5 are co-regulated in adult kidney (both increase in the Gv mutant) as they are in another mutant influencing ionic homeostasis in larval kidney [13].

In summary our results suggest that a member of the fifth class of G proteins, Gv is involved in regulating several developmental processes, with ionic homeostasis as a possible unifying theme. Often, but not always, Gv is expressed in the affected organs, suggesting that both direct action within a tissue and long-range effects exist. In the adult zebrafish, effects on ionic homeostasis - possibly modulated by compensatory processes – persist, as shown by our analysis of ion transporters in the adult kidney. This parallels high expression levels of Gv in adult kidney [5]. Future studies will be required to identify the molecular path by which Gv influences expression of ion transporter genes, as currently neither receptor(s) activating Gv nor downstream signalling molecules activatable by Gv are known. Nevertheless, our study represents the first step towards elucidating the function of an entire class of G alpha proteins.

### Electronic supplementary material

Below is the link to the electronic supplementary material.

Supplementary Material 1

## Data Availability

All study data are included in the article and/or SI materials.
