# Supplementary material for: Deciphering the function of the fifth class of Gα proteins: regulation of ionic homeostasis as unifying hypothesis
Source: Cell Mol Life Sci. 2024 May 10;81(1):213. doi: 10.1007/s00018-024-05228-w (PMC11087313; doi:10.1007/s00018-024-05228-w)
Supplement: Supplementary file 1 — Supplementary Material 1 [file 18_2024_5228_MOESM1_ESM.pdf]

Supporting Information for

Deciphering the function of the fifth class of Gα proteins: Regulation of ionic homeostasis as unifying hypothesis.

Asmaa Abu Obaid, Ivan Ivandic, and Sigrun I. Korsching

corresponding author: Sigrun I. Korsching
Email: sigrun.korsching@uni-koeln.de

This PDF file includes:

Figures S1 to S5 with legends
Tables S1 to S2
SI References

Table of Contents:

Figure S1	Negative control shows specificity of the *in situ* hybridization signal.
Figure S2	Gv expression at 72 hpf and Gv mutant cartilage and bone defects at 72 hpf and 11 dpf.
Figure S3	CRISPR/Cas9 targeting: analysis of Gv target site and potential off-targets with T7 endonuclease I
Figure S4	High levels of *gnav1* expression in oocytes.
Figure S5	Total volume and body weight are not altered in mutant larvae compared to wildtype.
Table S1	Relative expression levels of ionic homeostasis-related genes in Gv mutant zebrafish measured by qPCR.
Table S2	List of primers used for cloning for full-length *gnav1*, CRISPR/Cas9, amplification of in vitro transcription templates for *in situ* hybridisation, genotyping, and qPCR.
SI References	

Figure S1

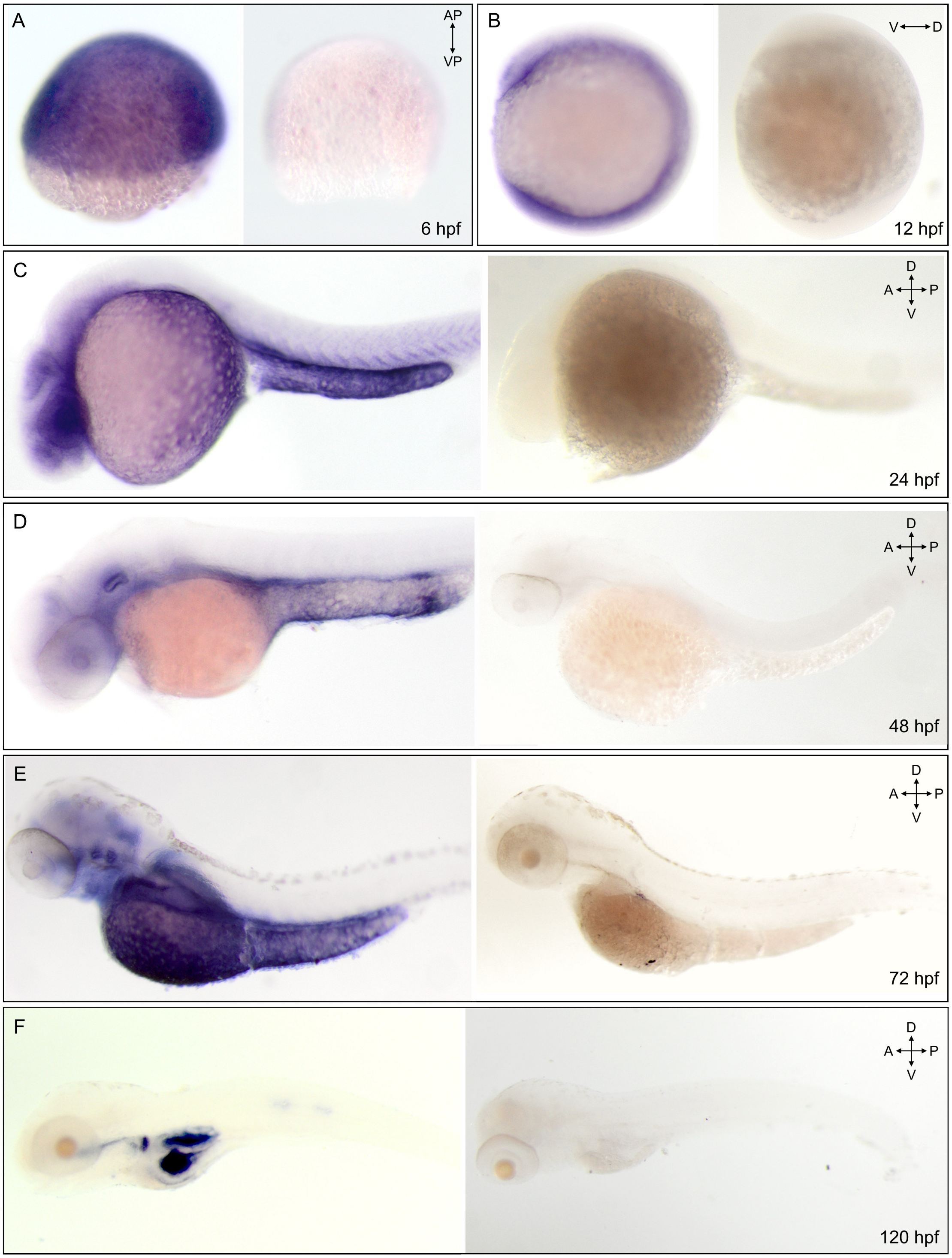
A
AP
VP
6 hpf
B
V
D
12 hpf
C
D
A
P
V
24 hpf
D
D
A
P
V
48 hpf
E
D
A
P
V
72 hpf
F
D
A
P
V
120 hpf

Figure S1 Negative control shows specificity of the *in situ* hybridization signal

For whole mount *in situ* hybridization sense probes were employed as negative control for each developmental stage. For each stage (enclosed by black frame) the antisense probe is shown to the left of the respective sense probe. Note the complete absence of signal with the sense probe, confirming the specificity of the signals obtained with antisense probes for *gnav1*. A) Developmental stage 6 hpf. B) 12 hpf. C) 24 hpf. D) 48 hpf. E) 72 hpf. F) 120 hpf.

Figure S2

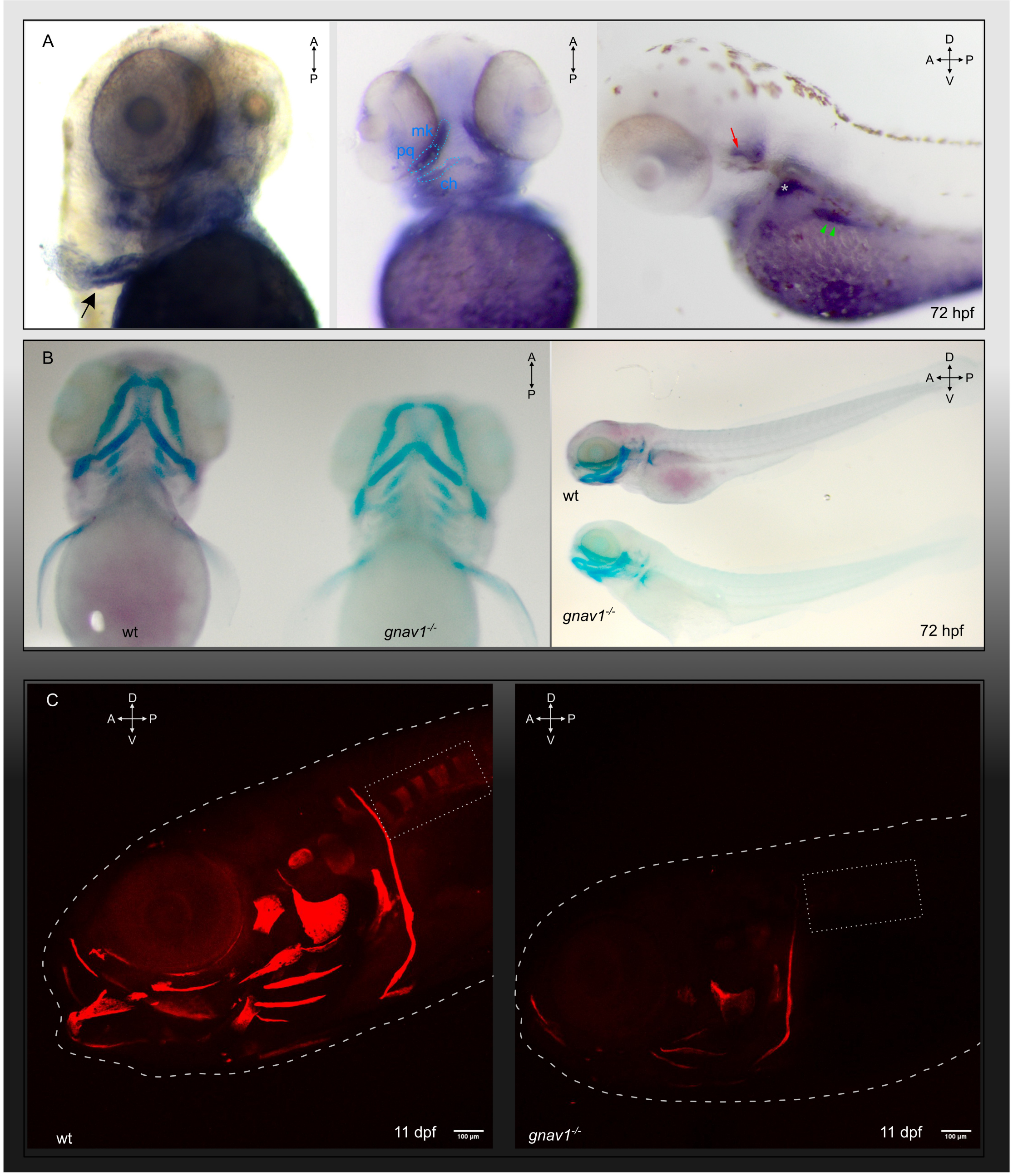
A
A
P
A
P
mk
pq
ch
D
A
P
V
72 hpf
B
A
P
wt
gnav1-/-
D
A
P
V
wt
gnav1-/-
72 hpf
C
D
A
P
V
wt
11 dpf
D
A
P
V
gnav1-/-
11 dpf

Figure S2 Gv expression at 72 hpf and Gv mutant cartilage and bone defects at 72 hpf and 11 dpf

A) Gv expression in wildtype 72 hpf larvae (brightfield images), orientation as indicated (A, anterior; P, posterior, D, dorsal; V, ventral). Left panel, two stripes of gnav1 expression in the actinotrichia of the pectoral fin. Middle panel, three cartilage regions expressing Gv can be distinguished: pq, palatoquadrate; mk, Meckel's cartilage; ch, ceratohyal. Right panel, asterisk, pronephros convoluted tubule (PCT); green arrowheads, gut; red arrow, anterior macula.B) Alcian Blue and ARS staining in 72 hpf larvae (brightfield images), orientation as indicated. Mutant larvae are to the left or below wildtype. Alcian Blue shows severely reduced staining in the mutant. Note that no bones have formed yet at this stage, thus only generalized ARS staining is visible in the wildtype, whereas no such staining is seen in the mutant. C) ARS staining in 11 dpf larvae (fluorescence shown), orientation as indicated. Left panel, wildtype; right panel mutant. Pictures were taken with identical settings. The dashed lines mark the body outline. Vertebrae are enclosed by dotted rectangles. Note that the reduced staining in the mutant is not limited to craniofacial bone structures, but extends to the vertebrae.

Figure S3

a

	Sequence	Score	Mismatches	Genomic position
Off-target 1	5'-TGTCTCAGGGGTGACAACAG-3'	1.6	[1:3:9]	chr5:66864257
Off-target 2	5'-GCACACAGAAGTGACAACAG-3'	1.3	[2:3:5:10]	chr23:31671507
Off-target 3	5'-GTGGTGAAAGGTGACAACAG-3'	0.8	[2:4:6:8]	chr14:40106835
Gv target	5'-GGGCTCAGAGGTGACAACAG-3'	89	/	chr22:11594399

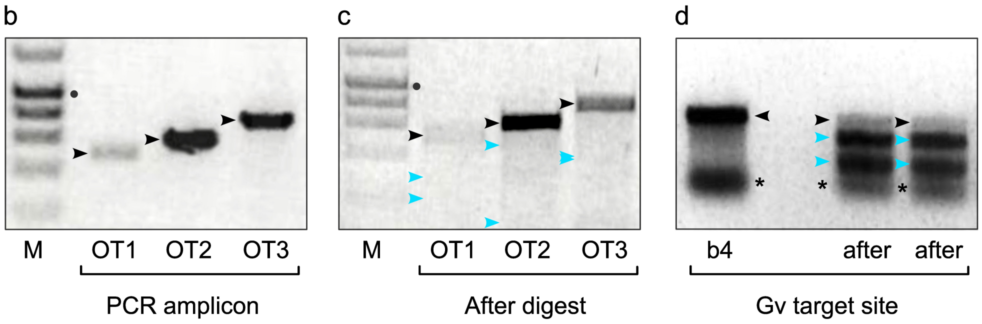

Figure S3 CRISPR/Cas9 targeting: analysis of Gv target site and potential off-targets with T7 endonuclease I

A) To predict potential off-target sites in the mutant, we employed an algorithm [1] as implemented on http://crispr.mit.edu/. The selected gRNA possessed only low quality potential off-target sites with 3 or more mismatches. Sequences of the three top-scoring potential off-target sites are shown with score and chromosomal location, mismatched bases are shown in red color, the Gv target sequence is shown below the line for comparison.

B) All three potential off-target sites were amplified from genomic DNA of the heterozygous F1 generation. M, marker lane, visible bands 1600, 1000 (marked by black dot), 800, 600, 400, 200; OT1, off-target 1; OT2, off-target 2; OT3, off-target 3; black arrowheads, bands correspond to predicted lengths for the uncut PCR amplicons.

C) Aliquots of PCR amplicons shown in B) were digested for 1 h at 37°C with 5 units T7 endonuclease I. Note the absence of fragments, expected position marked by blue arrowheads. Other labeling as in panel B).

D) For comparison the PCR amplicon for the Gv target site is shown before (b4) and after digest (two different F0 embryos) with T7 endonuclease I. Asterisks depict primer dimer. Note after digest the pronounced decrease in intensity for the uncut band (black arrowheads) and the appearance of two fragment bands (blue arrowheads) at the expected size. Together these results show the efficiency and specificity of the selected Gv target site.

Figure S4

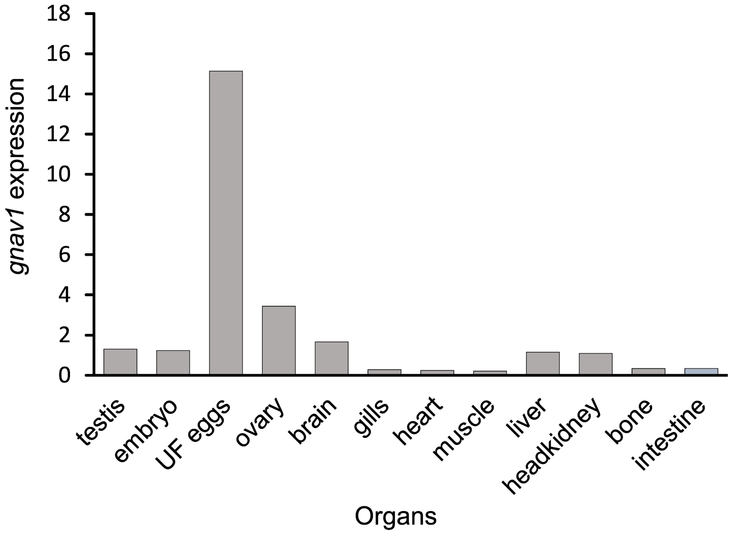

Figure S4 High levels of *gnav1* expression in oocytes

Expression levels were determined as rpkm (number of reads per kilobase per million reads, mean of 5 biological replicates) and normalized to the five reference genes (rpl8, b2m, eef1, actb1, rpl37) used in our qPCR experiments. Values shown represent geometric means. Note that unfertilized (UF) eggs, i.e. oocytes show the highest expression by far. The data used in this figure have been extracted from an RNA-Seq *de novo* analysis on 48157 contigs of zebrafish, which is part of the Phylofish project [2]. Data are accessible here: http://phylofish.sigenae.org/index.html

Figure S5

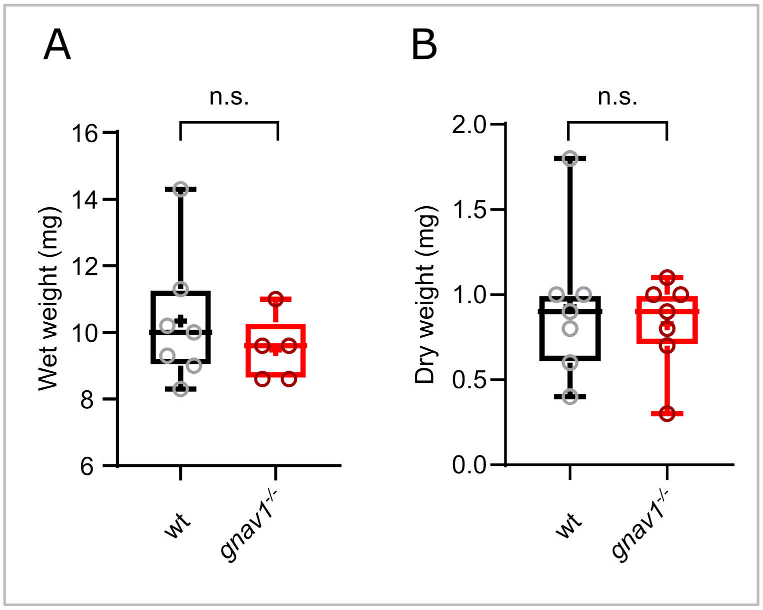

Figure S5: Total volume and body weight are not altered in mutant larvae compared to the wildtype

(A) the wet weight (before drying the larvae) was measured in pools of 25 larvae (5 dpf), n= 7 biological replicates. Note that there is no significant difference observed. (B) the dry weight was measured after drying the larvae overnight at 50 °C in pools of 25 larvae (5 dpf), n= 5-7 biological replicates. There is no significant difference in the dry weight between the wildtype and mutant larvae produced from siblings. n.s.; not significant.

Table S1. Relative expression levels of ionic homeostasis-related genes in Gv mutant zebrafish

				Kidney		3 dpf larvae		5 dpf larvae	
protein	gene name	location in cell	Ionocyte	fold change	p value	fold change	p value	fold change	p value
**Carbonic Anhydrase 2**	***ca2***	C	HR	1.52	0.24	n.d.		1.02	0.83
**ECaC**	***ecac***	A	NaR	1.58	0.37	0.90	0.45	1.49	0.11
**NCC**	***slc12a10.2***	A	NCC	7.48	0.51	n.d.		n.d.	
**NCC**	***slc12a3***	A	NCC	1.64	0.045	n.d.		1.66	0.52
**NCX1b**	***ncx1b***	B	NaR	2.30	0.041	n.d.		0.77	0.0486
**NHE3**	***slc9a3.2***	A	HR	1.94	0.11	n.d.		1.21	0.13
**NKA.1**	***atp1a1a.1***	B	NaR, SLC26	1.08	0.62	n.d.		1.78	0.21
**NKA.2**	***atp1a1a.2***	B	NCC	5.07	0.11	1.08	0.87	1.23	0.81
**NKA.4**	***atp1a1a.4***	B	KS	1.50	0.17	n.d.		0.86	0.30
**NKA.5**	***atp1a1a.5***	B	HR	3.47	0.007	0.61	0.015	0.94	0.56
**pendrin / SLC26**	***slc26a4***	A	SLC26	0.57	0.046	not expressed		1.01	0.96

Protein names are given in the 1st column; 2nd column, gene names. Full names are as follows: ECaC, epithelial calcium channel; NCC, thiazide-sensitive Na^+-Cl^- cotransporter; NCX1b, Na^+/Ca^{2+} exchanger; NHE, Na^+/H^+ exchanger 3; NKA, Na^+/K^+ ATPase transporting subunit alpha 1a; pendrin, Cl^-/HCO_3^- exchanger. Third column, location of protein in cell is indicated by A (apical), B (basal), and C (cytosolic). Ionocyte subtypes expressing these genes are given in the 4th column: HR, H^+ ATPase-rich ionocyte; KS, K^+-secreting ionocyte; NaR, Na^+/K^+ ATPase-rich ionocyte; NCC, Na^+-Cl^- cotransporter-expressing ionocyte; SLC26, solute carrier family 26-expressing ionocyte, location of proteins according to [3]. Results for adult kidney, 3 and 5 dpf larvae are shown. Sample size was one kidney, and pools of 20 larvae for 3 and 5 dpf. Five (rarely 4) biological replicates were examined for kidney, 5-6 biological replicates for 3 dpf, and 5 biological replicates for 5 dpf, each in two technical replicates; for method details see Materials and Methods section in main text. Significant changes in expression level are marked by green overlay.

Table S2. List of primers used for cloning for full-length gnav1, CRISPR/Cas9, amplification of in vitro transcription templates for in situ hybridisation, genotyping, and qPCR.

gnav1-EcoR1-F	TAAGCAGAATTCAATGGGTCTGTGTTTGGGCTC	cloning
gnav1-HindIII-R	TGCTTAAAGCTTGAGCAGAGAAACAGCCTCCAG	cloning
oligo1	TAGGGCTCAGAGGTGACAACAG	CRISPR/Cas9
oligo2	AAACCTGTTGTCACCTCTGAGC	CRISPR/Cas9
gnav1-F	TCACTCGGTTCTCTCGTGTG	In situ hybridisation
gnav1-R	AATGGTGTCCATCACCACCT	In situ hybridisation
gnav1-F3	CTGTGTTTGGGCTCAGAGGT	In situ hybridisation
gnav1-R3	TGTGGTTCTCAGTCGCACTC	In situ hybridisation
Ex1Gv-F	ACAAATGTCCGCGTCTTCTG	Genotyping
Ex1Gv-R	CGACCAATGTAAACATCAAAATC	Genotyping
atp1a1a.5-F	GTGGTTCTGTGCCTTCCCAT	qPCR
atp1a1a.5-R	CGGGTGTTCATTTTGATGTT	qPCR
b2m-F	GCCTTCACCCCAGAGAAAGG	qPCR
b2m-R	GCGGTTGGGATTTACATGTTG	qPCR
bactin-F	GCCTTCCTTCCTGGGTATGG	qPCR
bactin-R	CAGACGGAGTATTTACGCTCAG	qPCR
ca2-F	AGGACGCAGTTGATAAGCCT	qPCR
ca2-R	TGGACTTGATAGCATCCATA	qPCR
ECaC-F	TCCTTTCCCATCACCCTCT	qPCR
ECaC-R	GCACTGTGGCAACTTTCGT	qPCR
elf1-F	CTTCTCAGGCTGACTGTGC	qPCR
elf1-R	CCGCTAGCATTACCCTCC	qPCR
Gv-QPCR-Ex1-4-F	TCCATCTATGAATCGCCTGCAG	qPCR
Gv-QPCR-Ex1-4-R	GAACATGTCGGCGATGAAGC	qPCR
Gv-QPCR-Ex6-9-F	CGAGTACGCCAAGAGAGAGTT	qPCR
Gv-QPCR-Ex6-9-R	CCGAGTGAGCGTGAACCTT	qPCR
HRslc9a3.2-F	GCGAAACCCACCCTGGCAAAC	qPCR
HRslc9a3.2-R	GGCGAAGGAGTCTGTGGAGCG	qPCR
KSatp1a1a.4-F	TTCTGCCACTTCTGCCTTCC	qPCR
KSatp1a1a.4-R	CACCTTGATTCCAGCACTCC	qPCR
NarAtp1a1a.1-F	GCCCTGAGCAATTAGACGATG	qPCR
NarAtp1a1a.1-R	TACGGCTACAATGGCACCCT	qPCR
NCCatp1a1a.2-F	TCTACCTTTGGGCACCGTCAC	qPCR
NCCatp1a1a.2-R	TGCTTGGATCATCCCGATTT	qPCR
NCCslc12a3-F	CGATGATGGCGGTTTGACAC	qPCR
NCCslc12a3-R	TGAAACCCAGACGGAACCTG	qPCR
Ncx1b-F1	ATTGAGGCCATTACGGTCAG	qPCR
Ncx1b-R1	GATTGACACCACGAAACACG	qPCR
rpl37-F	ATGACGAAGGGTACGTCG	qPCR

rpl37-R	TTATGAGGAGCTGGACGC	qPCR
rpl8-F	CCGAGACCAAGAAATCCAGAG	qPCR
rpl8-R	CCAGCAACAACACCAACAAC	qPCR
slc12a10.2-F	GACCCAAGGTGGAGAGGACG	qPCR
slc12a10.2-R	CAGTTGATACCGATACTCAGC	qPCR
Slc26a4-F	TTCATTCGGATAGGGGTCAG	qPCR
Slc26a4-R	AAGAGAATGGCGTCGTGAAC	qPCR

Left column, primer name consists of gene name and F, forward or R, reverse direction. Middle column, primer sequences, given in 5'-3' direction. Right column, methods.

SI References

1. Hsu PD, Scott DA, Weinstein JA, et al (2013) DNA targeting specificity of RNA-guided Cas9 nucleases. Nat Biotechnol 31:827–832. https://doi.org/10.1038/nbt.2647

2. Pasquier J, Cabau C, Nguyen T, et al (2016) Gene evolution and gene expression after whole genome duplication in fish: the PhyloFish database. BMC Genomics 17:368. https://doi.org/10.1186/s12864-016-2709-z

2.

3. Guh Y-J, Lin C-H, Hwang P-P (2015) Osmoregulation in zebrafish: ion transport mechanisms and functional regulation. EXCLI J 14:627–659. https://doi.org/10.17179/excli2015-246
